# Implementation of quantum and classical discrete fractional Fourier transforms

**DOI:** 10.1038/ncomms11027

**Published:** 2016-03-23

**Authors:** Steffen Weimann, Armando Perez-Leija, Maxime Lebugle, Robert Keil, Malte Tichy, Markus Gräfe, René Heilmann, Stefan Nolte, Hector Moya-Cessa, Gregor Weihs, Demetrios N. Christodoulides, Alexander Szameit

**Affiliations:** 1 Institute of Applied Physics, Abbe School of Photonics, Friedrich-Schiller-Universität Jena, Max-Wien Platz 1, 07743 Jena, Germany; 2 Institut für Experimentalphysik, Universität Innsbruck, Technikerstraße 25, 6020 Innsbruck, Austria; 3 Department of Physics and Astronomy, University of Aarhus, 8000 Aarhus, Denmark; 4 INAOE, Coordinacion de Optica, Luis Enrique Erro No. 1, Tonantzintla, Puebla 72840, Mexico; 5 CREOL, The College of Optics & Photonics, University of Central Florida, Orlando, Florida 32816, USA

## Abstract

Fourier transforms, integer and fractional, are ubiquitous mathematical tools in basic and applied science. Certainly, since the ordinary Fourier transform is merely a particular case of a continuous set of fractional Fourier domains, every property and application of the ordinary Fourier transform becomes a special case of the fractional Fourier transform. Despite the great practical importance of the discrete Fourier transform, implementation of fractional orders of the corresponding discrete operation has been elusive. Here we report classical and quantum optical realizations of the discrete fractional Fourier transform. In the context of classical optics, we implement discrete fractional Fourier transforms of exemplary wave functions and experimentally demonstrate the shift theorem. Moreover, we apply this approach in the quantum realm to Fourier transform separable and path-entangled biphoton wave functions. The proposed approach is versatile and could find applications in various fields where Fourier transforms are essential tools.

## Abstract (web summary)

Fourier analysis has become a standard tool in contemporary science. Here, Weimann *et al*. report classical and quantum optical realizations of the discrete fractional Fourier transform, a generalization of the Fourier transform, with potential applications in integrated quantum computation.

## Graphical abstract

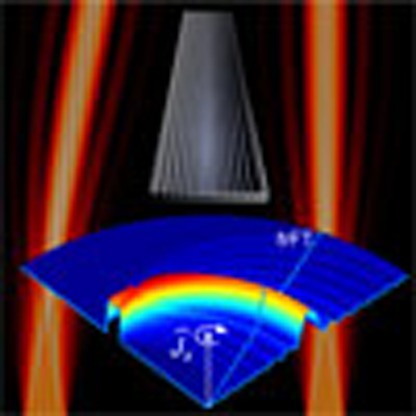

Two hundred years ago, Joseph Fourier introduced a major concept in mathematics, the so-called Fourier transform (FT). It was not until 1965, when Cooley and Tukey developed the ‘fast Fourier transform' algorithm, that Fourier analysis became a standard tool in contemporary sciences^1^. Two crucial requirements in this algorithm are the discretization and truncation of the domain, where the signals to be transformed are defined. These requirements are always satisfiable, since observable quantities in physics must be well behaved and finite in extension and magnitude.

In 1980, Namias made another significant leap with the introduction of the fractional Fourier transform (FrFT), which contains the FT as a special case^2^. Several investigations quickly followed, leading to a more general theory of joint time-frequency signal representations^3^ and fractional Fourier optics^4^. The vast scope of the FrFT has been demonstrated in areas such as wave propagation, signal processing and differential equations^3^,^5^,^6^,^7^. So far, the FT of fractional order was realized only by single-lens systems^8^,^9^, although other theoretical suggestions, including multi-lens systems^10^ or graded index fibres exist^11^. The aim to discretize this generalized FT led to the introduction of the discrete fractional Fourier transform (DFrFT) operating on a finite grid in a way similar to that of a discrete FT^12^. Along those lines, several versions of the DFrFT have been introduced^5^, however, without any experimental realization, so far. In this work we focus on the optical implementation of the so-called Fourier–Kravchuk transform^12^ that can be equally applied to the classical and quantum states. Throughout our paper, we simply refer to this transform as DFrFT whose application reaches from the demonstration of the Fourier suppression law^13^, N00N-state generation^14^,^15^ and qubit storage^16^ to the realization of perfect discrete lenses for non-uniform input distributions.

In this work, we report on the realization of DFrFTs of one-dimensional optical signals based on an integrated lattice of evanescently coupled waveguides. In these photonic arrangements, the inter-channel couplings are designed in such a way that the system readily performs the DFrFT of any incoming signal. The signal evolution is governed by the Schrödinger equation and the associated Hamilton operator is known as the *J*_*x*_-operator in the quantum theory of angular momentum or likewise as the Heisenberg *XY* model from the quantum theory of ferromagnetism. We foresee that the inherent versatility of this approach will make other realizations of the DFrFT, the FT and the fast Fourier transform recognizably simple and thus may open the door to many interesting applications in integrated quantum computation^17^.

## Results

### Theoretical approach

**Figure 1:**
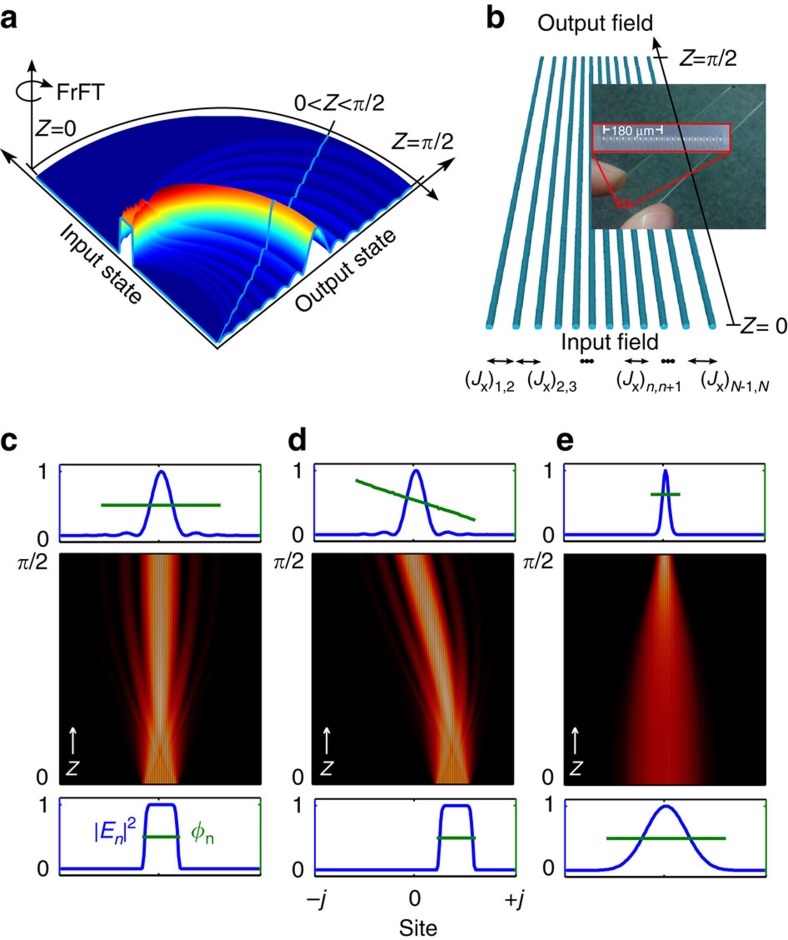
Discretization of the FrFT. (**a**) Pictorial view of actual fractional Fourier transforms exemplified as continuous rotations in phase space. (**b**) Schematic representation of a pre-engineered *J*_*x*_-array. (**c**–**e**) Top views of continuous ‘rotations' of a rectangular (**c**), displaced rectangular (**d**) and Gaussian (**e**) optical wave functions in a *J*_*x*_-array with *N*=151. The bottom and top plots show the intensities of the ingoing and outgoing wave packets, respectively. The green lines describe the magnitude of phase distributions of the optical fields, that is, the phase jumps of *π* due to a change of sign of the signal's amplitude are not shown.

Similarly to its continuous counterpart, the DFrFT can be interpreted physically as a continuous rotation of the associated wave functions through an angle *Z* in phase space (see Fig. 1a)^18^. The idea is thus to construct finite circuits that are capable of imprinting such rotations to any light field. In quantum mechanics, three-dimensional spatial rotations of complex state vectors are generated via operations of the angular momentum operators *J*_*k*_ (*k*=*x*, *y*, *z*) on the Hilbert space of the associated system^19^. In particular, the rotation imprinted by the *J*_*x*_-operator turns out to be an elaborated definition of the DFrFT (see Methods section for discussion). These concepts can be readily translated to the optical domain by mapping the matrix elements of the *J*_*x*_-operator over the inter-channel couplings of engineered waveguide arrays (Fig. 1b–e)^20^. The coupling matrix of such waveguide arrays is thus given by 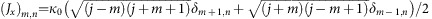 (ref. 19). Here, *κ*_0_ is a scaling factor introduced for experimental reasons. The indices *m* and *n* range from −*j* to *j* in unit steps. Meanwhile, *j* represents an arbitrary positive integer or half-integer that determines the total number of waveguides via *N*=2*j*+1 (Fig. 1b).

Coupled mode theory states that the evolution of light in the *J*_*x*_-waveguide array is governed by the following set of equations^20^


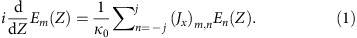


Here, *E*_*n*_(*Z*) denotes the complex electric field amplitude at site *n*. In the quantum optics regime, single photons traversing such devices are governed by a set of Heisenberg equations that are isomorphic to equation (1). The only difference is that in the quantum case *E*_*n*_(*Z*) must be replaced by the photon creation operator 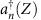. In a spintronic context, the evolution parameter *Z* is associated with time, whereas in the framework of integrated quantum optics, *Z* represents the propagation distance, see Fig. 1b–e. A spectral decomposition of the *J*_*x*_-matrix yields the eigenvectors 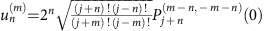
14,19, which in combination with the eigenvalues, 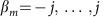, render the closed-form point-spread function


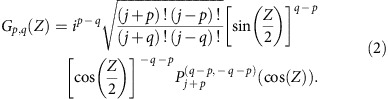


Note that *q* and *p* represent the excited and observed sites, respectively, and 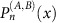 are the Jacobi polynomials of order *n* (see Methods section for discussion). Using equation (2), we can compute the response of the system to any input signal, which in turn gives the DFrFT^12^. Accordingly, DFrFT of any particular order arises at one specific propagation distance *Z* lying between 0 and *π*/2. In the limit *N*→∞, the eigenvectors of *J*_*x*_, 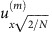, become the continuous Hermite–Gauss polynomials *H*_*m*_(*x*), which are known to be the eigenfunctions of the fractional Fourier operator^12^. As a result, in the continuous limit, the DFrFT described by equation (2) converges to the continuous FrFT^5^,^12^; and the standard FT is recovered at *Z*=*π*/2 (Fig. 1c–e). Note that in general, the DFrFT obtained in our devices and the usual DFT become equal only in the continuous limit *N*→∞ and at *Z*=*π*/2.

### Experiments with classical light

**Figure 2:**
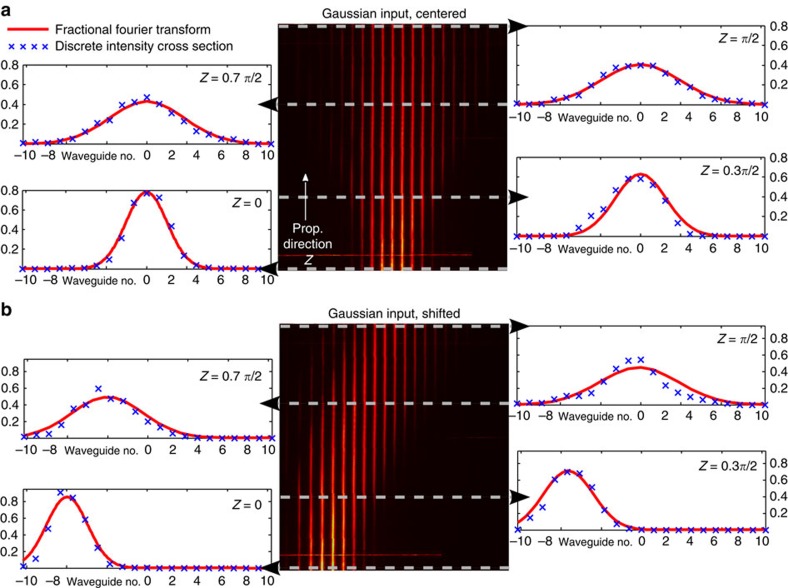
DFrFT of classical light. (**a**) Transformation of a Gaussian input into a Gaussian profile of larger width along the evolution in the *J*_*x*_-array. The FT is obtained at *Z*=*π*/2. The experimental data (blue crosses) is compared with the numeric FrFT (red curves). (**b**) A shifted input Gaussian profile evolves towards the centre of the array and acquires the same width as in **a**.

To experimentally demonstrate the functionality of the suggested waveguide system, we use *N*=21 waveguides to perform FTs of simple wave packets. We first consider a Gaussian wave packet with a full-width at half-maximum (FWHM) covering the five central sites (Fig. 2a). The input signal is prepared by focusing a Gaussian beam from a HeNe laser onto the front facet of the sample. By exploiting the fluorescence from colour centres within the waveguides^21^, we monitor the full intensity evolution from the input to the output plane. The fluorescence image, Fig. 2a, shows a gradual transition from an initially narrow Gaussian distribution at the input to a broader one at the Fourier plane (left and right panels Fig. 2a), demonstrating that narrow signals in space correspond to broad signals in Fourier space. For intermediate propagation distances (*Z*∈[0, *π*/2]) we extract other orders of the DFrFT, simultaneously. For comparison, we plot the continuous FrFT produced by the corresponding continuous Gaussian profile (red curves Fig. 2a). The agreement between the computed FrFT and the experimental DFrFT proves that for the considered Gaussian input signal, *N*=21 is sufficient to achieve the continuous limit. We now shift the input Gaussian beam by six channels towards the edge. Since the separations between adjacent waveguides at the edges are bigger than the separations between adjacent waveguides in the centre, the discretization grid is not perfectly homogeneous. Strictly speaking, the discretized shifted Gaussian just at the input plane covers slightly less than five waveguides FWHM. We observe that the well-approximated off-centre Gaussian travels to the centre at *Z*=*π*/2 (Fig. 2b), hereby showing the famous shift theorem. In additional experiments, extended signals, for example, a shifted top-hat function, are found to be well transformed according to equation (2) as well. However, we find that for this type of excitation *N*>21 would be required to discuss the continuous limit (see Supplementary Note 1 with Supplementary Fig. 1).

**Figure 3:**
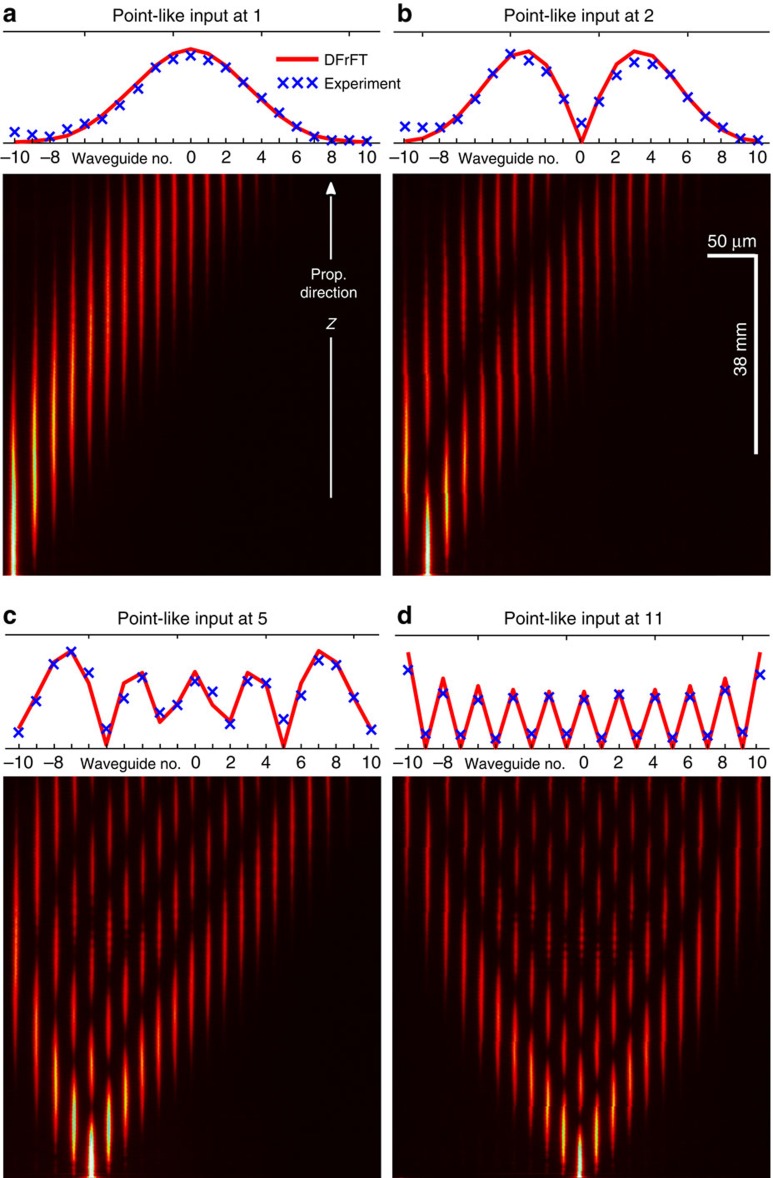
Experimental visualization of the discrete Hermite–Gauss polynomials. (**a**–**d**) Evolution of single-site inputs into the magnitudes of the respective eigensolutions, as predicted theoretically (methods). The experimental data (blue crosses) is compared with the analytic DFrFT (red curves).

An unequivocal criterion, for the functionality of devices that perform the DFrFT, equation (2), can be formulated by evaluating 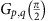. At this particular distance, point-like excitations will give rise to signal magnitudes that perfectly resemble the magnitudes of one of the eigenstates of the transform. More specifically, for transforms such as equation (2), one finds that an excitation of the *q*th site excites the *q*th system eigenstate up to local phases 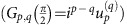 (see the Methods section for explanations). The experimental demonstration of this intriguing effect is shown in the subpanels of Fig. 3a–d along with the theoretical predictions. It can be argued that for any point-like excitation, the continuous limit cannot be met experimentally (see the Methods for discussion). Instead, equation (2) creates a non-uniform amplitude distribution with a phase difference of *π*/2 between adjacent sites. Nevertheless, in the continuous limit, 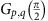 tends to the usual FT kernel^12^. At this point, it is worth emphasizing the formal equivalence to the quantum Heisenberg *XY* model in condensed matter physics^22^,^23^. In this respect, our observations demonstrate the capability of the here-presented systems to store quantum information in *XY* Hamiltonians by converting specific inputs into eigenstates of the system^16^. To our knowledge, this rather rare property has never been thoroughly investigated before.

### Quantum experiments

To demonstrate the applicability of our approach in the quantum domain, we now analyse intensity correlations of separable and path-entangled photon pairs propagating through these Fourier transformers. To do so, we fabricated *J*_*x*_-arrays involving *N*=8 channels. The importance of exploring FTs of such states has been highlighted in several investigations, demonstrating interesting effects such as suppression of states and portraying biphoton spatial correlations^24^,^25^,^26^.

**Figure 4:**
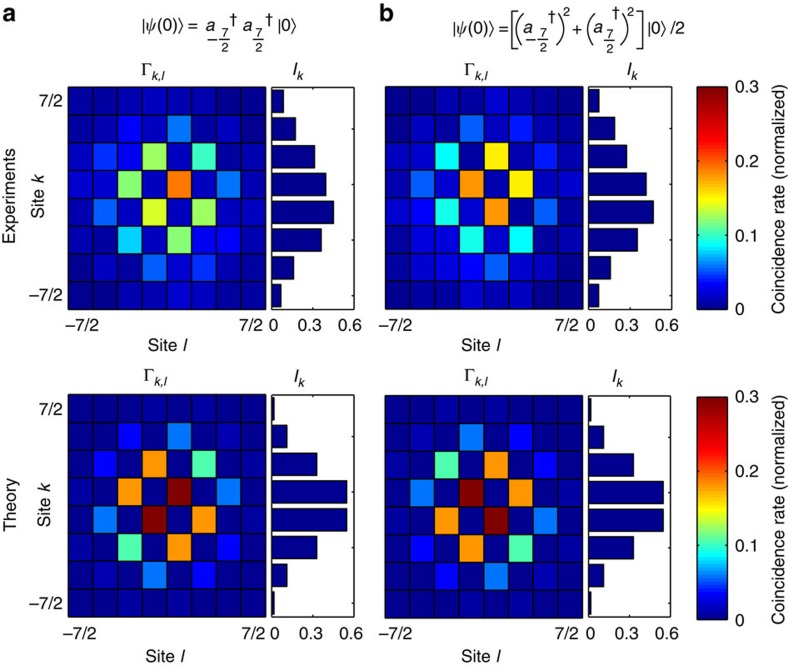
DFrFT of quantum light. Correlation maps Γ_*k*,*l*_ of a two-photon state either prepared (**a**) in a product state or (**b**) in a path-entangled state after propagating through a *J*_*x*_-array. The photon density *I*_*k*_ at the output is shown on the right side of each map. The evaluation of the s.d. of the coincidence rates is presented in the Supplementary Note 2.

In this discrete quantum optical context, pure separable two-photon states are readily produced by coupling pairs of indistinguishable photons into two distinct lattice sites (*m*, *n*), this state is mathematically described by 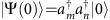. Conversely, path-entangled two-photon states are created by simultaneously launching both photons at either site *m* or *n* with exactly the same probability, that is, 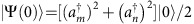. Furthermore, the probability of observing one of the photons at site *k* and its twin at site *l* is given by the intensity correlation matrix 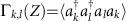 (ref. 27). An intriguing and unique property of the *J*_*x*_-systems is that at *Z*=*π*/2 the correlation matrices are given in terms of the eigenstates, as noticed above. Hence, for the separable case, 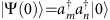, the correlation matrices are given by 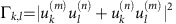, whereas for the path-entangled state, 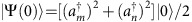, we have 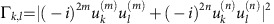. Of particular interest is the separable case, where the photons are symmetrically coupled into the outermost waveguides, 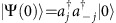. In this scenario, only the correlation matrix elements for which (*k*+*l*)=odd are nonzero, and are given by 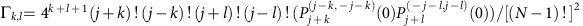. These effects are demonstrated for the initial state 
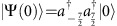
 in Fig. 4a, where concentration and absence of probability in the correlation matrix clearly show that some states are completely suppressed—a hallmark of any Fourier unitary process^13^. An estimation of the statistical significance of the data set, along with a short discussion on incoherence effects can be found in the Supplementary Note 2 involving Supplementary Figs 2 and 3.

**Figure 5:**
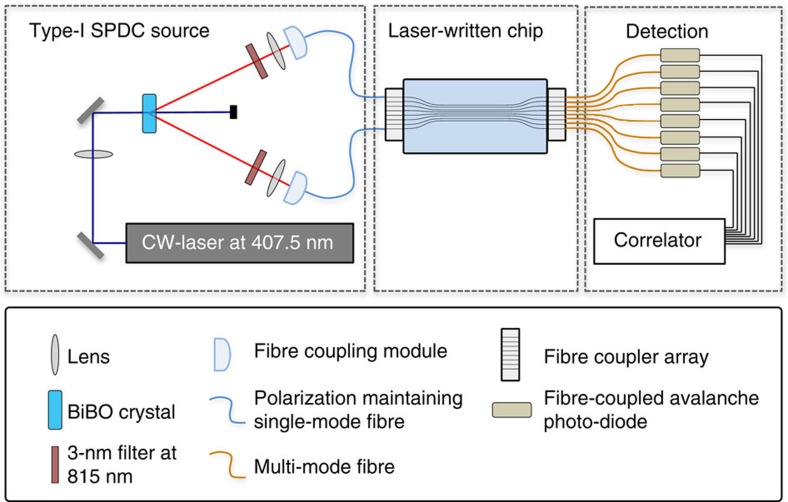
Set-up used to carry out spatial correlation measurements of photonic quantum states. The set-up consists of three parts—the state preparation, the execution of the DFrFT and the correlation measurement.

As a second case, we consider a fully symmetric path-entangled two-photon state of the form 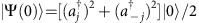. Physically, both photons are entering together into the array at either site *j* or −*j* with equal probability^28^,^29^,^30^. The correlations are determined by 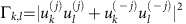, from which we infer that the probability of measuring photon coincidences at coordinates (*k*, *l*) vanishes at sites where the sum (*k*+*l*) is odd. In contrast, at coordinates where (*k*+*l*) is even, the correlation function collapses to the expression 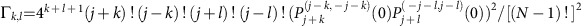. This indicates that in this path-entangled case the correlation map appears rotated by 90° with respect to the matrix obtained with separable two-photon states. We performed an experiment to demonstrate these predictions using states of the type 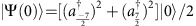, which were prepared using a 50:50 directional coupler acting as a beam splitter^29^. The whole experiment is achieved using a single chip containing both the state preparation stage followed by a *J*_*x*_-system, yielding high interferometric control over the field dynamics (Fig. 5). The experimental measurements are presented in Fig. 4b. Similarly, suppression of states occurs as a result of destructive quantum interference. As predicted, a closer look into the correlation pattern reveals that indeed the correlation map appears rotated by 90° with respect to the matrix obtained with separable two-photon states.

## Discussion

We emphasize that our quantum measurements feature interference fringes akin to the ones observed in quantum Young's two-slit experiments of biphoton wave functions in free space as demonstrated in ref. 24. In such free-space experiments, however, far-field observations were carried out using lenses and the two slits were emulated by optical fibres^24^. Along those lines, we have created a fully integrated quantum interferometer to observe fundamental quantum mechanical features^25^. This additionally suggests an effective way to generate quantum states containing only even (odd) non-vanishing inter-particle distance probabilities for the separable input state (symmetric path-entangled state). In addition, the eigenfunctions associated with the Hamiltonian system explored in our work are specific Jacobi polynomials, which are well known as the optimal basis for quantum phase-retrieval algorithms^31^ and these eigenstates can be retrieved by limited phase operations. Knowing a quantum wavefunction and its FT, a phase-retrieval algorithm for signals that are a superposition of a finite number of Hermite–Gauss polynomials has been introduced^32^. This phase-retrieval algorithm might be implemented employing our system, since its discrete character automatically possesses a finite number of polynomials that are closely connected to the Hermite–Gauss polynomials. Another potential application is the realization of the Radon–Wigner transform given by the squared modulus of the FrFT^6^,^33^. The Radon–Wigner function is a basic tool for the reconstruction of Wigner quasi-probability distributions in quantum optics^34^,^35^. Also, FrFTs appear naturally in optics as free-space propagation between two spherical reference planes in general^4^. Like in our system, the order of the FrFT is proportional to the propagation distance. On that basis, complex spatial filtering involving several fractional Fourier planes was suggested^36^. In this description, the optical signal is discretized and thus described by a vector of field amplitudes at certain sites. In our approach this is inherently realized.

In conclusion, we have successfully demonstrated a universal discrete optical device capable of performing classical and quantum DFrFTs. Our studies might find applications in developing a more general quantum suppression law^13^ and perhaps in the development of new quantum algorithms.

## Methods

### The fractional Fourier transform in quantum harmonic oscillations

In this section, we briefly describe the relation between the continuous FrFT operator and the Hamiltonian of the quantum harmonic oscillator^2^.

The FrFT operator 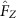 is defined by the following eigenvalue equation involving the Hermite–Gauss polynomials of order *n* and the eigenvalues *λ*_*n*_=exp(*inZ*)


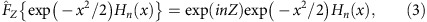


where 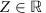. Concurrently, one can interpret equation (3) as quantum time evolution from time *t*=0 to *t*=*Z*. We write 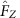 as 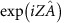.


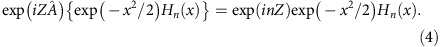


To show that 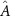 is the Hamilton operator of the harmonic oscillator, we differentiate both sides of equation (4) with respect to *Z* and evaluate the result at *Z=*0. We obtain


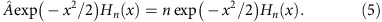


To find the spatial representation of 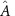, consider the differential equation


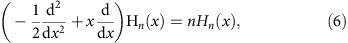


for the Hermite polynomials *H*_*n*_(*x*) of order *n*. Using the identities 1=exp(*x*^2^/2)exp(−*x*^2^/2) and 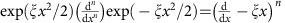, one can show that equation (6) can be written as


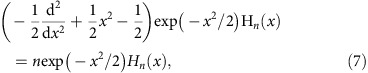


where we have used the commutator 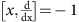. Comparing equation (5) and equation (7), one can see that 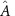 becomes


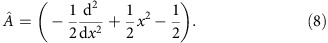


In summary, the FrFT operator 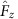 can be written as


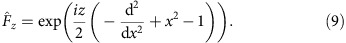


Because of this one-to-one correspondence between the dynamics of the quantum harmonic oscillator and the fractional Fourier operator, the implementation of such transform is immediate using harmonic oscillator systems^37^.

### *J*_*x*_-photonic lattices as discrete harmonic oscillators

Our aim in this section is to show that in the continuous limit *N*→∞, the eigenvalue equation for *J*_*x*_-arrays becomes the eigenvalue equation of the quantum harmonic oscillator. Consider the matrix representation of the *J*_*x*_-operator again. For convenience we take, in this section only, *κ*_0_=1.


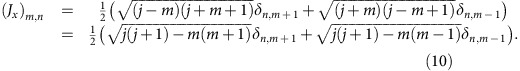


The indices *m* and *n* range from −*j* to *j* in unit steps and *j* is an arbitrary positive integer or half-integer. The dimension of the *J*_*x*_-matrix is *N*=2*j*+1. We now introduce the variable *γ*=*j*(*j*+1)=(*N*^2^−1)/4, which implies that (*J*_*x*_)_*m*,*n*_ can be written as


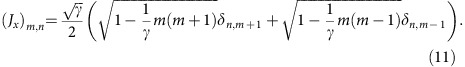


Let us consider the eigenvalue equation for this matrix


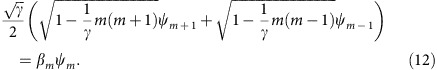


Considering the region 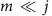, since *γ*∝*N*^2^, in the limit *N*→∞, the terms 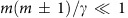. Hence, in the domain far from the edge of the array a Taylor expansion yields


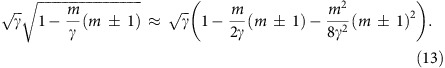


By defining *m*=*xγ*^1/4^ (or *x*=*m/γ*^1/4^) , we obtain


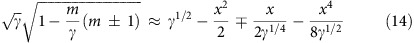


Plugging this expression into equation (12)


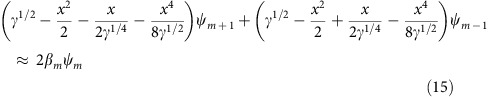


We redefine the functions 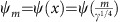 and 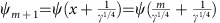 such that we can introduce the Taylor series


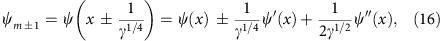


where, again, we have kept only terms up to second order in 1/*γ*^1/4^. Substituting equation (16) into equation (15), and using the limit


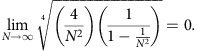


We obtain the time-independent Schrödinger equation for the harmonic oscillator


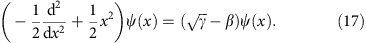


Therefore, in the continuous limit *N*→∞, the difference equation describing *J*_*x*_-photonic lattices becomes the time-independent Schrödinger equation for the quantum harmonic oscillator. Note, however, that due to the importance of the condition 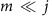 in this derivation, this statement is only valid when dealing with signals that are square integrable in the continuous limit. Thus, the operator equation (11) can be used to define the discrete version of the quantum harmonic oscillator and thus the DFrFT.

### The point-spread function for *J*_*x*_-photonic lattices

In this section, it is shown that at *Z*=*π*/2, the green function of *J*_*x*_-systems becomes proportional to the amplitude of one of the eigenstates. The evolution of light in *J*_*x*_-arrays is governed by the set of *N* coupled differential equations (equation (1)).

The normalized propagation coordinate *Z* is given by *Z*=*κ*_0_*z*, where *z* is the actual propagation distance and *κ*_0_ is an arbitrary scale factor. The quantity *E*_*n*_(*Z*) denotes the mode field amplitude at site *n*. A spectral decomposition of the *J*_*x*_-matrix yields the eigensolutions


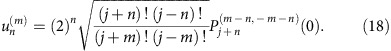



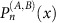
 are the Jacobi polynomials of order *n*. And the corresponding eigenvalues are integers or half-integers, *β*_*m*_=−*j*, …, *j*, depending on the parity of *N*^14^,^19^. Using the eigenvectors and eigenvalues we obtain the point-spread function


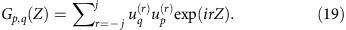


*G*_*p,q*_(*Z*) represents the amplitude at site *p* after an excitation of site *q*. Using equation (18) and the properties of the Jacobi polynomials one can show that equation (19) reduces to the closed-form expression


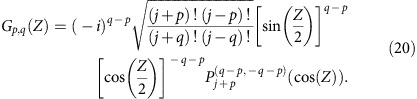


Evaluation of equation (20) at *Z*=*π*/2 yields


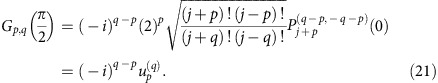


Equation (21) shows that at *Z*=*π*/2 the point-spread function becomes proportional to the amplitude of the corresponding eigenstates depending on the excited site. In other words, there is a one-to-one correspondence between the excited site number and the eigenstates of the system: excitation of the *q*th site excites the *q*th eigenstate up to well-defined local phases.

### Devices fabrication and specifications

Our devices are fabricated in bulk-fused silica samples (Corning 7980, ArF grade) using the femtosecond laser direct-write approach^21^. The transparent material is modified within the focal region due to nonlinear absorption resulting in a local increase of the refractive index. Effectively, the waveguides possess only the fundamental mode. The coupling between neighbouring waveguides depends on their separation within the glass chip. Regarding the theoretical description of the waveguide array, the validity of equation (1) is only given if the fundamental modes of neighbouring waveguides have a negligible overlap. For a given *N* and a maximum length of the glass wafers of *Z*/*κ*_0_, this can only be ensured for a certain range of κ_0._ Outside this range of *κ*_0_, coupled mode theory will break down and errors are introduced to the implementation of the DFrFT. In our experiments these errors are kept small but impossibly perfectly zero.

For the fabrication of the devices used to transform classical light, we employed an Yb-doped fibre laser (Amplitude Systèmes) operating at a wavelength of 532 nm, a repetition rate of 200 kHz and a pulse length of 300 fs. Waveguides were written with 300 nJ pulses focused by a 20x objective. The sample was moved at a velocity of 200 mm min^−1^ by high-precision positioning stages (ALS 130, Aerotech Inc.) with a positioning error of ±0.1 μm. From this random positioning error, the realized inter-channel couplings inherit a relative error of 2%. The mode field diameters of the guided mode were 4 μm × 7 μm at 632 nm. In the classical experiments, the Fourier plane lies at Z/*κ*_0_=7.48 cm, that is, *κ*_0_=0.21 cm^−1^. The desired nearest-neighbor couplings 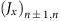
determine the separations of the waveguides *n* and *n*±1. The largest separation of 22.8 μm between adjacent waveguides occurs at the edges. In the centre, the separation is 17.6 μm.

For the samples illuminated with single-photon states of light, we used a RegA 9,000 seeded by a Mira Ti:Sa femtosecond laser oscillator. The amplifier produced 150 fs pulses centred at 800 nm at a repetition rate of 100 kHz, with energy of 450 nJ. The structures were permanently inscribed with a 20x objective while moving the sample at a constant speed of 60 mm min^−1^, using the positioning system described above. The mode field diameters of the guided mode were 18 μm × 20 μm at 815 nm. All structures were designed with fan-in and fan-out sections arranged in a three-dimensional geometry, and located prior and after the *J*_*x*_-lattice, as illustrated in Fig. 5. This effectively suppresses any unwanted crosstalk between the guides and permits easy coupling to fibre arrays with a standard spacing of 127 μm. In the presented device used to transform quantum states, we have *κ*_0_=0.6 cm^−1^, that is, the Fourier plane is located 2.62 cm after the beginning of the *J*_*x*_-array.

### Experiment on the characterization of two-photon correlations

A BiB_3_O_6_ nonlinear crystal was pumped with a 70 mW continuous wave pump laser emitting at 407.5 nm, which provided pairs of indistinguishable photons due to type-I spontaneous parametric down-conversion, see Fig. 5. Photon pairs with a central wavelength of 815 nm were filtered by 3 nm (FWHM) interference filters. They were further coupled to the chip via fibre arrays through polarization maintaining fibres, and subsequently fed into single-photon detectors (avalanche photodiodes). The two-photon correlation function was determined by analysing the twofold coincidences recorded between all output channels with the help of an electronic correlator card (Becker & Hickl: DPC230). The spatial correlation results presented in Fig. 4 were extracted from a data set with total integration time of 5 min. The coincidences were then analysed with a time window set at 5 ns and are corrected for detector efficiencies. To assess the statistical consistency of the results in Fig. 4, we discuss in the Supplementary Note 2 the data set presented in terms of correlation event numbers before normalization. For both measurements, the detector clicks data set initially consisted of ∼1.5 × 10^6^ events in total, which after post selection was reduced to a set of ∼5 × 10^4^ coincidence events in total, for both input states. Accidental coincidences due to simultaneous detection of two photons not coming from the same pair are estimated to occur with a negligible rate of <2 × 10^−6^s^−1^. Non-deterministic number-resolved photon detection was achieved using fibre beam splitters. This set-up thus allows the determination of all 36 two-photon coincidence events occurring in photonic lattices consisting of eight channels. Furthermore, at the wavelength of interest, propagation losses and birefringence are estimated to be 0.3 dB cm^−1^ and in the order of 10^−7^, respectively. Additional discrepancies in between the measured correlation matrices and the theoretical ones may appear due to imperfect excitations, asymmetric output coupling losses (or detector efficiencies) or limited indistinguishability of the photons.

## Additional information

**How to cite this article:** Weimann, S. *et al*. Implementation of quantum and classical discrete fractional Fourier transforms. *Nat. Commun.* 7:11027 doi: 10.1038/ncomms11027 (2016).

## Supplementary Material

Supplementary Information Supplementary Figures 1-3, Supplementary Notes 1 – 2 and Supplementary References.
