# Supplementary material for: Implementation of quantum and classical discrete fractional Fourier transforms
Source: Nat Commun. 2016 Mar 23;7:11027. doi: 10.1038/ncomms11027 (PMC4814576; doi:10.1038/ncomms11027)
Supplement: Supplementary Information — Supplementary Figures 1-3, Supplementary Notes 1 – 2 and Supplementary References. [file ncomms11027-s1.pdf]

Supplementary Information on

Implementation of Quantum and Classical Discrete Fractional Fourier Transform

Supplementary Figures

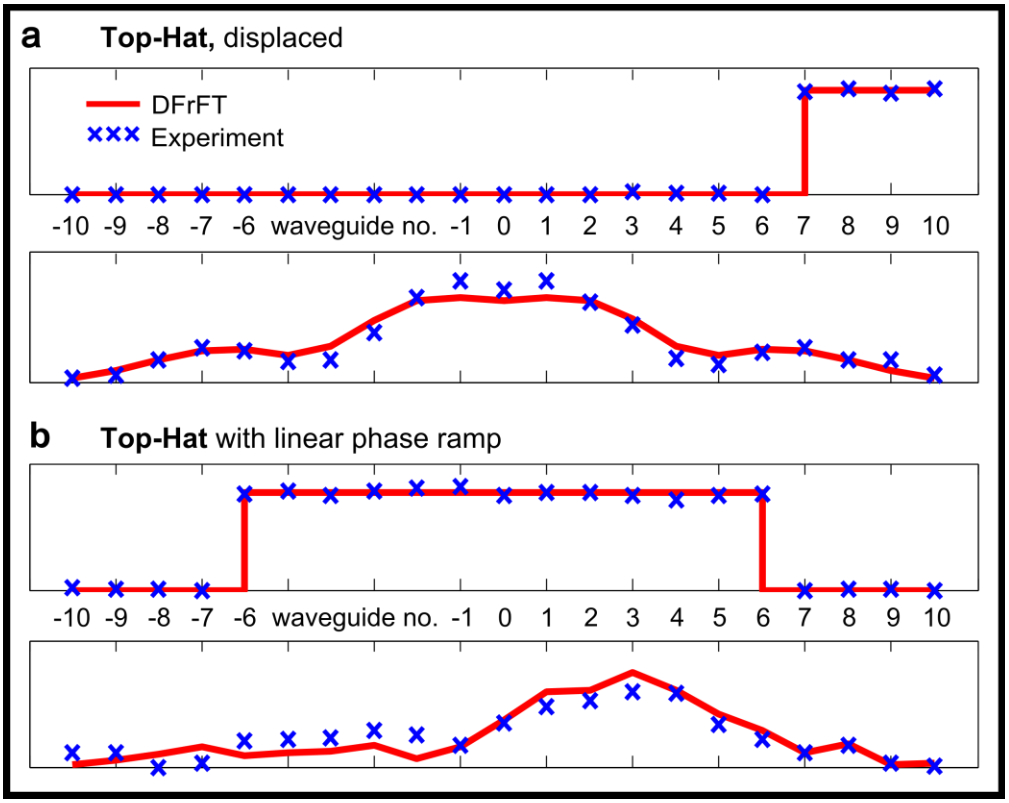

Supplementary Figure 1: Experimental observation of DFrFTs of top-hat functions (a) launched at the edge and (b) launched at the center (with a linear phase ramp) of a J_x-photonic lattice. Upper panels in a and b show the respective input intensity distributions while the lower panels show the output intensity distributions.

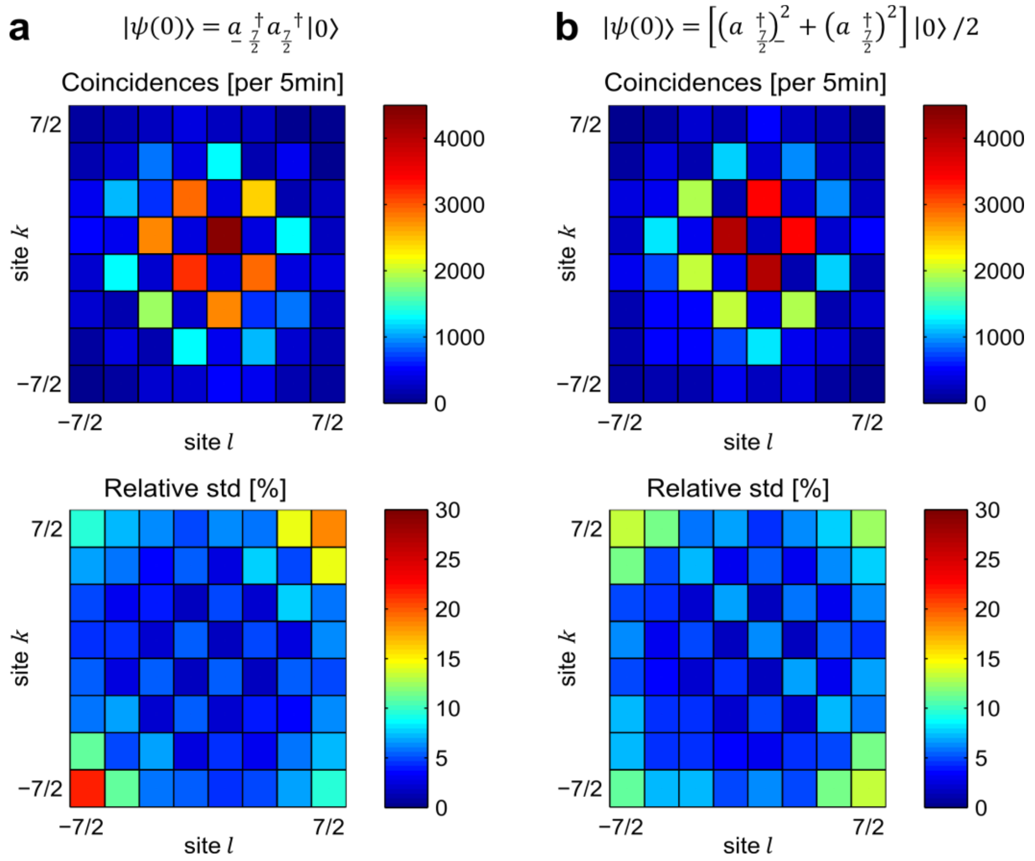

Supplementary Figure 2: Statistical consistency of the obtained quantum measurements (a) for the product state and (b) for the entangled state considered in the study. Top matrices show the same data set than Fig. 4 in the main text without normalizing as correlation function, that is, expressed in correlation event numbers (see colorbar). Bottom matrices show an estimator of the relative standard deviation (std.) of the measurement, expressed in percent (see colorbar).

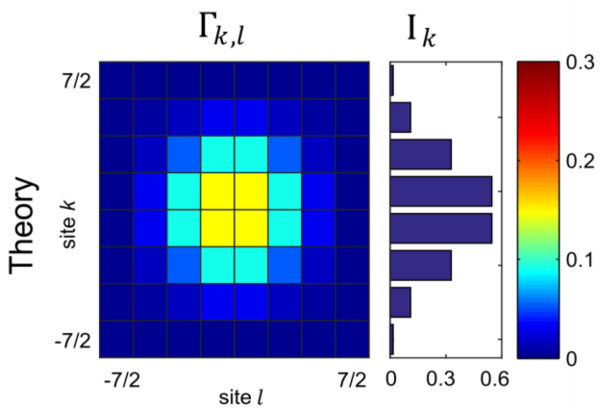

Supplementary Figure 3: Correlations of distinguishable photons Theoretical correlation maps $\Gamma_{k,l}$ for a pair of distinguishable photons after propagating through a J_x-lattice. The photon density I_k at the output is shown on the right side of the map.

Supplementary Note 1

As additional classical experiments we examine a well-known example of a FT pair, the top-hat function whose FT is a sinc function. This input state is intentionally chosen to illustrate the difference between the FT and the DFrFT when the continuous limit is not met. The input fields are prepared by tailoring laser light at a wavelength of 632 nm using a SLM (Holoeye Pluto VIS). In order to be able to shape the input field at will, we modulate amplitude and phase. Since this SLM allows for phase modulation only, the amplitude modulation is realized by imprinting an extra phase grating onto the SLM. The first diffraction order can thus be modulated in amplitude as well (1). An initial Gaussian beam at a wavelength of 632 nm is expanded to homogeneously illuminate the display of the SLM. The reflected beam is Fourier transformed by a spherical lens with 300mm focal length. The first diffraction order is isolated by a slit aperture 300mm after the lens. The so prepared field distribution is scaled down to micrometer size by means of a 4f configuration involving a 250mm lens and a 20x microscope objective (Olympus Plan Achromat). For the sake of maximizing the efficiency of coupling the free space electric field distribution into the discrete array of single-mode waveguides, the beams are prepared as arrays of narrow Gaussian spots matching the mode profile of the waveguides. Amplitudes and positions of all individual Gaussian spots are adjusted according to the desired input beam profile and the positions of the waveguides in the input plane. For examining the input, a CCD camera above the input plane is utilized to image the fluorescence emitted from the excited waveguides.

With a second camera behind the chip we record the intensity distribution, e.g. in the output plane. The sample itself is mounted on a rotation stage for controlling the tilt of the sample's front facet with respect to the phase front of the incident light.

In Supplementary Figure 1a, we show an initial top-hat pattern covering 4 waveguides at the edge of the array (top panel). We thus break the correspondence to the harmonic oscillator as explained at the end of section "J_x-photonic lattices as discrete harmonic oscillators

" in the Methods. There is no phase shift between adjacent sites. The angle between phase front and the sample's front facet is adjusted to zero by maximizing the incoupling efficiency of a single Gaussian spot being incident upon one of the waveguides. The adjustment of the input angle is checked by launching an extended Gaussian beam containing of 5 spots and verifying a zero-shift of the maximum between input and output plane. In the bottom panel of Supplementary Fig. 1a the measured output field is compared to the computed DFrFT. Although the agreement between the measurement in the J_x- lattice and the theoretical DFrFT is very good, one can clearly see deviations between the DFrFT and a sinc function. Furthermore, we launch a centered broader top-hat distribution with a linear phase ramp (Supplementary Figure 1B). This is achieved by tilting the array by an angle of approximately 0.01°. This very small tilt is not significantly influencing the input intensity distribution which is created by the 4f-setup. As can be seen, the ramping phase in the input field does not result in a pure spatial displacement at $Z = \pi/2$. Again there are deviations between the DFrFT and the shifted sinc function, meaning that the continuous limit is not achieved.

Supplementary Note 2

In order to assess the statistical consistency of the results in Fig. 4 of the main text, we show in Supplementary Figure 2 (top matrices) the same data set presented in correlation event numbers $C_{k,l}$. Assuming a Poissonian counting statistics, a good estimator of the standard deviation of this distribution can be given by its square root $\sqrt{C_{k,l}}$. We determine the relative standard deviation (std.) $\sqrt{C_{k,l}}/C_{k,l}$, and show it in percent in Supplementary Figure 2 (bottom matrices) for the corresponding input states. One can observe that typical values obtained lie in the range of a few percent for the more populated elements in the

correlation matrix and, naturally, are higher for elements with small population. The mean relative standard deviations are 5.8% and 5.9% for the product state and the entangled state measurements, respectively. This procedure therefore fully supports the statistical significance of the presented results in the main text.

In order to compare the results to a classical, incoherent counterpart, we have performed an additional simulation of the correlation function when the lattice is illuminated with distinguishable photons at the lattice edges. After injection into channels $\boldsymbol{k}$ and $\boldsymbol{l}$, the correlation function of a two-photon wavefunction with distinguishable particles reads $\boldsymbol{\Gamma_{k,l}} = \left|\boldsymbol{U_{m,k}U_{n,l}}\right|^{\mathbf{2}} + \left|\boldsymbol{U_{m,l}U_{n,k}}\right|^{\mathbf{2}}$. The simulation result is shown below. Such correlation map does not show any signature of quantum interference since time-evolved states of this kind can always be factorized. As a result, the building-up of non-classical correlations is prevented. Evidently, the correlation maps obtained either with the product state or the entangled state in the main text (Fig. 4) show a checkerboard pattern, as states are suppressed due to destructive interference between the different paths each photon can take. This is in strong contrast to the simulation presented here.

Supplementary References

[1] J. A. Davis, D. M. Cottrell, J. Campos, M. J. Yzuel and I. Moreno, Encoding amplitude information onto phase-only filters, Appl. Opt. **38**, 5004-5013 (1999)
